# Megacolon Associated with Multiple Endocrine Neoplasia Type 2B: A Case Successfully Managed with Ileostomy

**DOI:** 10.70352/scrj.cr.26-0202

**Published:** 2026-05-02

**Authors:** Takeshi Ikeda, Nao Hondo, Masato Kitazawa, Satoshi Nakamura, Yuta Yamamoto, Satoru Miyazaki, Masahiro Kataoka, Hirokazu Tanaka, Naoki Ishizaka, Tadaaki Shimizu, Masatsugu Kuroiwa, Naoya Yamamoto, Yuji Soejima

**Affiliations:** Department of Gastroenterological, Transplantation and Pediatric Surgery, Shinshu University School of Medicine, Shinshu University Hospital, Matsumoto, Nagano, Japan

**Keywords:** MEN2B, megacolon, ganglioneuromatosis, ileostomy, colostomy

## Abstract

**INTRODUCTION:**

Multiple endocrine neoplasia type 2B (MEN2B) is a rare genetic disorder characterized by medullary thyroid carcinoma, pheochromocytoma, mucosal neuromas, and Marfanoid body habitus. Megacolon is a common non-endocrine manifestation, with approximately 75% of patients experiencing gastrointestinal symptoms, such as constipation or diarrhea, often as early clinical signs. Although these symptoms may be important diagnostic signs, there are currently no distinct therapeutic guidelines. Here, we report a case of MEN2B-associated megacolon that was managed surgically, resulting in a favorable outcome.

**CASE PRESENTATION:**

A 23-year-old man with a history of chronic constipation was diagnosed with MEN2B at 14 years of age based on findings of medullary thyroid carcinoma, mucosal neuromas, and rectal biopsy-confirmed intestinal ganglioneuromatosis. He previously underwent total thyroidectomy and emergency transverse colectomy for volvulus. Progressive colonic dilatation led to abdominal distension and organ compression, prompting referral. Imaging confirmed marked colonic dilation. Laparoscopic subtotal colectomy with ileosigmoid anastomosis was performed. Postoperatively, the patient experienced recurrent ileus and required hospital readmission for small bowel obstruction. Two months postoperatively, the patient presented with a gastrointestinal perforation at the anastomotic site and underwent resection with terminal ileostomy. The postoperative course was uneventful, and the patient remained symptom-free.

**CONCLUSIONS:**

MEN2B-associated megacolon results from diffuse intestinal ganglioneuromatosis. Considering its progressive and extensive nature, decompression via stoma creation may be necessary to improve the postoperative outcomes and reduce the risk of symptom recurrence. Increased awareness and individualized surgical planning are crucial for treating this rare but serious complication of MEN2B.

## Abbreviation

MEN2B
: multiple endocrine neoplasia type 2B

## INTRODUCTION

MEN2B is a rare genetic disorder, with an estimated incidence of 1.4–2.6 cases per 1000000 individuals.^1–3)^ A hallmark feature is the inevitable development of medullary thyroid carcinoma, with approximately 50% of patients developing pheochromocytoma. Megacolon is a common non-endocrine manifestation, with approximately 75% of patients presenting with gastrointestinal symptoms, such as constipation or diarrhea, which are often observed as early clinical signs.^4)^ These symptoms are increasingly recognized as potential indicators for early diagnosis; however, clear therapeutic guidelines remain unestablished. Here, we report a case of MEN2B in which an ileostomy was performed to address severe gastrointestinal manifestations, resulting in a favorable postoperative course.

## CASE PRESENTATION

A 23-year-old man (height: 1.71 m; weight: 51 kg; BMI: 17 kg/m^2^) presented with progressive abdominal distension. He had a history of chronic constipation since childhood and complained of abdominal distension due to refractory megacolon. A rectal biopsy confirmed intestinal ganglioneuromatosis, raising a suspicion of MEN2B. A pathogenic variant in the *RET* gene was identified. and the patient was diagnosed with MEN2B. Additionally, medullary thyroid carcinoma and mucosal neuromas of the tongue and lips were identified, and a total thyroidectomy was performed. Subsequently, the patient developed transverse colon volvulus and underwent emergency transverse colectomy.

**Fig. 1:**
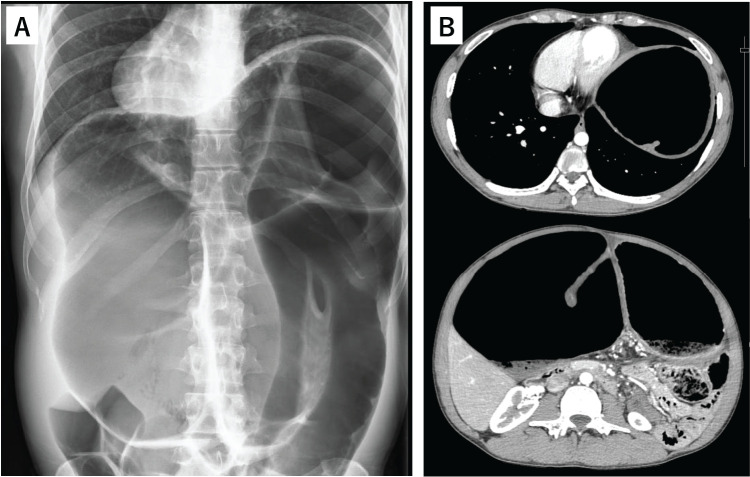
Imaging findings at initial presentation. (**A**) Abdominal radiography shows diaphragm elevation, with rightward displacement of the heart. (**B**) Enhanced CT shows marked colonic dilatation, with rightward displacement of the heart.

Progressive colonic dilatation caused diaphragm elevation and compression of adjacent organs. At 23 years of age, he was referred to our department for surgical management. He was taking levothyroxine and Bifidobacterium. No pheochromocytomas or paragangliomas were observed. The abdomen was markedly distended but soft and non-tender with a midline surgical scar. Laboratory test results revealed no abnormalities. Abdominal radiography revealed marked colonic dilatation with diaphragmatic elevation and rightward displacement of the heart (**Fig. 1A**). These findings were confirmed using contrast-enhanced CT (**Fig. 1B**).

**Fig. 2:**
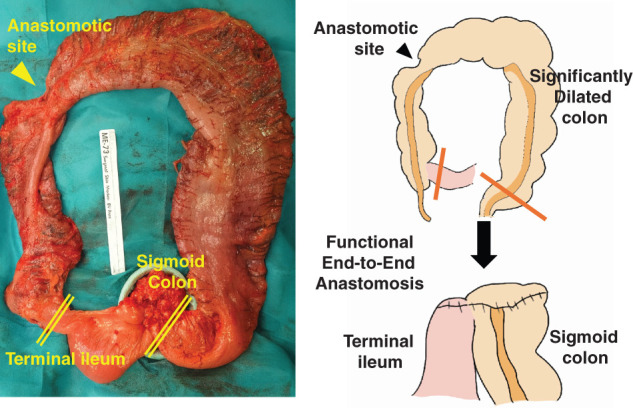
Intraoperative image of the ileum to the colon. The anastomotic site is indicated by an arrowhead, and the colon was markedly dilated. A subtotal colectomy was performed, and the colon was anastomosed using a functional end-to-end anastomosis.

**Fig. 3:**
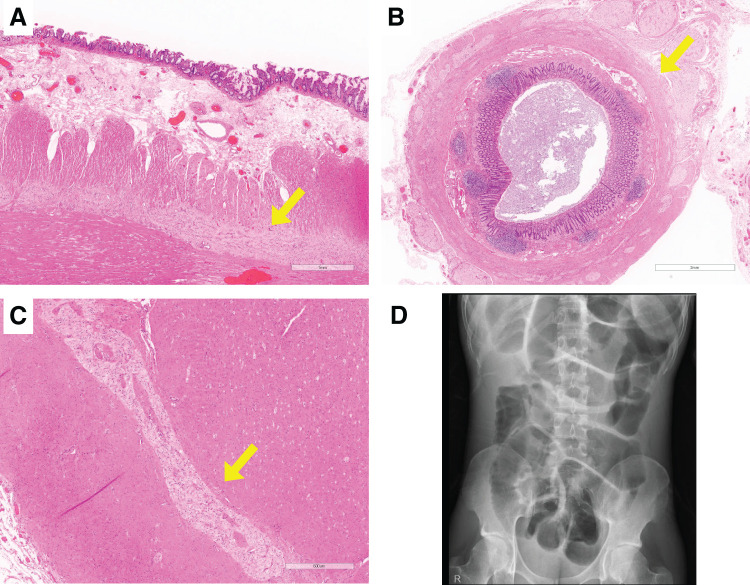
Histopathological findings. (**A**) Small intestine. (**B**) Appendix. (**C**) Colon. All of these show hyperplasia of ganglion cells and nerve fibers (arrow). (**D**) Radiography upon hospital discharge after subtotal colectomy. The small intestine dilation persisted.

**Fig. 4:**
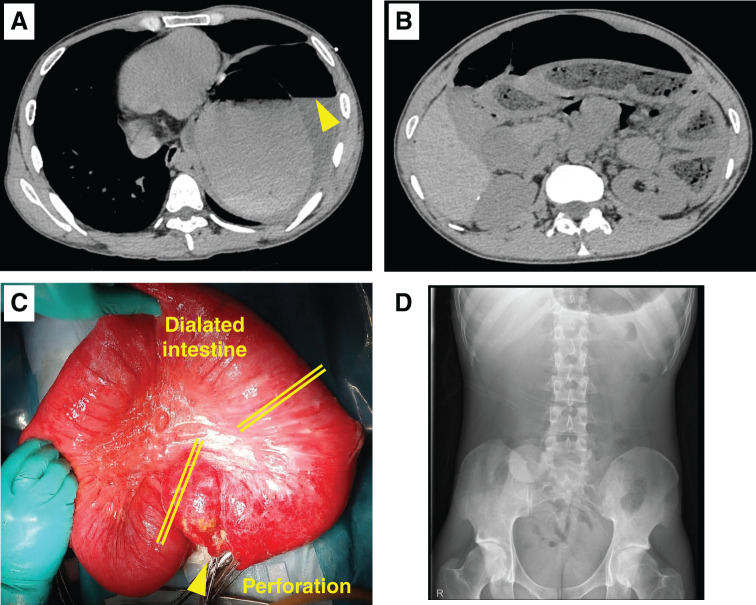
(**A**, **B**) On emergency admission, CT reveals a large amount of free air with associated ascites (arrowhead). (**C**) A perforation at the anastomotic site (arrowhead) required a partial bowel resection and the creation of an ileostomy. (**D**) On the pre-discharge radiography following ileostomy surgery, abnormal bowel gas had disappeared.

Laparoscopic-assisted subtotal colectomy (cecum to the descending colon) was performed for the MEN2B-associated megacolon. The remaining part of the colon was severely dilated (**Fig. 2**). The dilated colon was resected, and the ileum and sigmoid colon were anastomosed using a functional end-to-end anastomosis. The procedure was completed without major complications (operative time: 152 min; blood loss: 10mL). Histopathological examination revealed prominent myenteric plexus layers in the colon, small intestine, and appendix, with hyperplasia of the ganglion cells and nerve fibers (**Fig. 3A**–**3C**). These findings were consistent with those of intestinal ganglioneuromatosis. Oral intake was resumed on POD 3; however, on POD 5, the patient developed vomiting, and radiographic examination revealed ileus. Despite long intestinal tube insertion, drainage was inadequate, suggesting pseudo-obstruction. Ileus symptoms improved on POD 10, allowing tube removal and diet resumption; however, the symptoms recurred on POD 16. Although abdominal radiography continued to show intestinal gas retention (**Fig. 4D**), the patient’s symptoms improved, and he was discharged on POD 22.

One month after discharge, the patient was readmitted with recurrent ileus, which was conservatively managed using a long intestinal tube. After 2 months, the patient presented with acute abdominal pain following a meal and was urgently transported to our hospital. CT revealed massive free air and ascites consistent with gastrointestinal perforation (**Fig. 4A** and **4B**), prompting an emergency laparotomy. The residual small intestine was markedly dilated, and a perforation was identified at the previous anastomotic site (**Fig. 4C**). A perforated segment resection and terminal ileostomy were performed. The postoperative course was uneventful. Follow-up imaging revealed no abnormal intestinal gas retention (**Fig. 4D**), and the patient was discharged on POD 9. The patient remained symptom-free for 10 months postoperatively.

## DISCUSSION

MEN is a group of autosomal dominant hereditary syndromes characterized by tumors in multiple endocrine organs and classified into MEN1 and MEN2.^5)^ Among these, MEN2B is an extremely rare subtype, accounting for approximately 5% of all MEN2 cases, with an estimated incidence of 1.4–2.6 cases per 1000000 individuals.^1–3)^ The condition is caused by mutations in the RET protooncogene.^6,7)^ A hallmark feature of MEN2B is the inevitable development of medullary thyroid carcinoma, and approximately 50% of patients develop pheochromocytoma.^8,9)^ Additional clinical manifestations include mucosal neuromas, intestinal ganglioneuromatosis, and a Marfanoid body habitus.^10,11)^ Approximately 75% of patients with MEN2B experience gastrointestinal symptoms, such as constipation and diarrhea, which often appear early and serve as important diagnostic clues.^4)^ However, the therapeutic strategies for these symptoms remain unestablished. Megacolon arises from intestinal ganglioneuromatosis, characterized by the hyperplasia of ganglion cells and nerve fibers throughout all layers of the intestinal wall, unlike Hirschsprung disease, which is characterized by aganglinosis.^12)^ This results in impaired peristalsis and progressive bowel dilatation. Approximately one-third of affected patients require surgical intervention^11,13)^; however, there are currently no standardized guidelines for the surgical management of MEN2B-associated megacolon.

Currently, 13 cases of MEN2B with surgically treated megacolon have been reported (**Table 1**).^11,13–17)^ Of these, 9 patients initially underwent bowel resection with primary anastomosis, whereas 4 underwent stoma creation. Among those who underwent resection and anastomosis, symptoms recurred in 5 patients (55.6%), and 4 of them (44.4%) required stoma formation. One patient showed improvement with conservative treatment, including pelvic floor muscle training. Of the 8 patients who ultimately underwent stoma creation, 6 had a favorable postoperative course. Contrastingly, 2 patients experienced poor outcomes: one developed worsening symptoms after colostomy and underwent colostomy revision and ileostomy, whereas the other encountered difficulties in managing stoma output and electrolyte balance, resulting in a prolonged hospital stay of 62 days. Notably, both patients had concomitant pheochromocytoma.^11,17)^

**Table 1:** Previous case reports of surgically treated megacolon for MEN2B and clinical courses

Case	Author	Initial surgery	Postoperative exacerbation	Additional treatment	Outcome and remarks
Case 1	Gibbons et al.^11)^	Subtotal colectomy	+	Ileostomy	Good progress
Case 2	Gibbons et al.^11)^	Total colectomy	+	Pelvic floor muscle training	Responded well
Case 3	Gibbons et al.^11)^	Colectomy + colostomy	+	Revision of colostomy Ileostomy	Responded well Concomitant pheochromocytoma
Case 4	Gibbons et al.^11)^	Subtotal colectomy	−	−	Good progress
Case 5	Cohen et al.^13)^	Small bowel resection	+	Colectomy + colostomy	Responded well
Case 6	Cohen et al.^13)^	Colectomy	−	−	Good progress
Case 7	Cohen et al.^13)^	Small bowel resection + appendectomy	−	−	Good progress
Case 8	Cohen et al.^13)^	Small bowel resection + appendectomy	−	−	Good progress
Case 9	Iturrino et al.^14)^	Subtotal colectomy	+	Sigmoid resection + ileostomy Anti-ChE inhibitor	Responded well
Case 10	Fernando et al.^15)^	Total colectomy + ileostomy	−	−	Good progress
Case 11	Xu et al.^16)^	Subtotal colectomy + ileostomy	−	−	Good progress
Case 12	Wang et al.^17)^	Subtotal colectomy +	+	Managed medically	Suspected concomitant pheochromocytoma
Case 13	Our case	Subtotal colectomy	+	Sigmoid resection + ileostomy	Good progress

ChE, cholinesterase; MEN2B, multiple endocrine neoplasia type 2B

These findings suggest that megacolon in MEN2B results from diffuse intestinal ganglioneuromatosis, and bowel resection with primary anastomosis alone may be insufficient to control the symptoms. Conversely, several reports indicate that decompression via stoma creation can lead to favorable postoperative outcomes.^11,13,15)^ An ileostomy may offer a more reliable, long-term solution for patients with intractable or recurrent symptoms. In cases of recurrence or poor outcomes despite stoma creation, the possibility of hypercatecholaminemia due to undiagnosed pheochromocytoma should be carefully considered.

MEN2B is rare and has a poor prognosis, resulting in the limited availability of long-term follow-up data. Further accumulation of cases with extended observations is warranted to establish optimal management strategies.

## CONCLUSIONS

Megacolon associated with MEN2B arises from intestinal ganglioneuromatosis, which frequently affects the entire gastrointestinal tract. Considering the diffuse nature of this pathology, decompression via stoma creation can improve the postoperative outcomes and reduce the risk of symptom recurrence. Increased awareness and individualized surgical planning are crucial for treating this rare but serious complication of MEN2B.
